# Utilization of Bovine Pericardium Patch During Common Femoral Endarterectomy

**DOI:** 10.3390/jcm14113852

**Published:** 2025-05-30

**Authors:** Dóra Zoé Zatykó, Enikő Pomozi, Dániel Pál, Tamás Kovács, Zoltán Szeberin

**Affiliations:** Department of Vascular and Endovascular Surgery, Semmelweis University Heart and Vascular Centre, 1122 Budapest, Hungary

**Keywords:** femoral endarterectomy, bovine pericardium patch, surgical site infection, critical limb ischemia, patch angioplasty, arteriotomy closure

## Abstract

**Background/Objectives:** Common femoral endarterectomy (CFE) is one of the most frequent open arterial surgical procedures. The ideal material to close the arteriotomy is equivocal. This study aims to evaluate the efficacy and safety of bovine pericardium patch (BPP) utilization in femoral artery bifurcation endarterectomy (FE). **Methods**: A single-center, retrospective study was conducted, involving 200 consecutive FE procedures performed between November 2019 and December 2022. Clinical data, including demographics, comorbidities, surgical details, and outcomes, were collected from institutional records. The primary endpoints were overall survival, reintervention-free survival, and amputation-free survival. Secondary endpoints included the incidence of surgical site infection (SSI) and its associated risk factors. Logistic regression models were used to identify predictors of SSI, adjusting for confounders such as age, smoking, comorbidities, and bacterial colonization. **Results**: The median age of the cohort was 68 (SD ± 9.70) years, and 66% were male. The median follow-up period was 1010 (SD ± 471.47) days. Thirty-day survival was 91%, and 2 year survival was 69.3%, with cardiovascular events and cancer being the leading causes of death. Reintervention-free survival was 94.7% at 30 days and 77.5% at 2 years, while amputation-free survival was 94.3% at 30 days and 87.4% at 2 years. SSI requiring surgery occurred in 16% of patients, with a higher risk observed in patients with critical limb ischemia (CLI) compared to those with claudication. The presence of pathogens such as MRSA, *Escherichia coli*, and *Pseudomonas aeruginosa* (OR 16.1, *p* < 0.001) was significantly associated with SSI. Previous groin surgery did not affect SSI incidence. **Conclusions**: BPP utilization in FE provides favorable patency and survival outcomes, even in a high-risk patient population with significant comorbidities. CLI and bacterial colonization increased the risk of SSI. Perioperative infection prevention strategies and management of systemic comorbidities are essential to improve patient outcomes.

## 1. Introduction

Even in the era of advanced endovascular techniques, femoral endarterectomy (FE) remains a cornerstone in the surgical treatment of atherosclerotic lesions of the common femoral artery (CFA). Open endarterectomy is considered a safe, durable, and cost-effective surgical procedure used mainly in chronic limb ischemia. Despite the increasing use of percutaneous interventions, open surgical endarterectomy continues to be the gold standard procedure when it comes to the revascularization of the CFA, demonstrating long-term patency rates ranging from 80% to 96% [1,2,3]. To optimize outcomes and prevent restenosis, patch angioplasty (PA) is frequently used following common femoral endarterectomy (CFE). Autologous vein patches (AVPs), traditionally favored for their biological compatibility, are not without limitations. Availability issues and the need to preserve the great saphenous vein for potential future bypass procedures have prompted the exploration of alternative materials. Furthermore, concerns regarding patch rupture and the relatively high-risk of infection associated with prosthetic patches, particularly in the groin region, necessitate careful consideration in the selection of patch material [4]. Surgical site infections (SSIs) are a critical outcome to monitor after surgery because they can significantly impact patient recovery, increase the risk of graft failure, prolong hospital stays, and lead to serious complications such as limb loss or sepsis.

Bovine pericardial patch (BPP) angioplasty has emerged as a promising alternative, offering durability and a lower risk of complications such as rupture and infection [5]. Recent work by Hostalrich et al. demonstrated promising mid-term outcomes using bovine pericardial tube grafts for emergency reconstruction in infected native and prosthetic femoral arteries, reporting high technical success and acceptable reintervention-free survival rates at 12 months [6]. While BPP has shown encouraging results in carotid and other vascular surgeries [7,8,9], its application in femoral endarterectomy requires further evaluation, particularly concerning infection rates, postoperative complications, mortality, and amputation-free survival. This study aims to assess the efficacy and safety of femoral endarterectomy with bovine pericardium patch angioplasty. We focus on evaluating early postoperative complications, infection rates, and long-term outcomes, including mortality and amputation-free survival, to determine whether BPP is a viable option for improving surgical outcomes in patients undergoing FE.

## 2. Materials and Methods

In a single-center retrospective study, we examined 200 cases over a 2 year period (from 1 November 2019 to 31 December 2022). All patients were diagnosed and treated at the Semmelweis University Heart and Vascular Centre, Department of Vascular and Endovascular Surgery, where they underwent CFE with bovine pericardium patch angioplasty (XenoSure^®^ Biologic Patch, LeMaitre Vascular Inc., Burlington, MA, USA) for ischemia or bleeding. Patients with pre-existing inguinal infections were excluded. All procedures analyzed in this study were unilateral. The primary endpoints were overall survival, reintervention-free survival, and amputation-free survival. The secondary endpoints were postoperative wound infection requiring surgery, factors influencing the occurrence of infection, the occurrence of septic rupture, and hospital death within 30 days.

Monitoring was based on clinical follow-up. For patients treated for claudication or bleeding, follow-up is determined on an individual basis, but the standard follow-up visit is usually scheduled at 3 months. For patients undergoing surgery for rest pain or ischemic tissue loss, the usual follow-up visit is scheduled at our outpatient clinic after 1 month. An ankle brachial index is performed at this visit. Further visits are scheduled on an individual basis depending on the wound healing process or the severity of symptoms. In the event of inadequate wound healing, worsening symptoms, or suspicion of decreased patency, additional imaging is performed, including ultrasound, CTA or DSA.

Surgical site infection (SSI) requiring surgery was diagnosed based on clinical symptoms and microbiological testing, following the Centers for Disease Control and Prevention (CDC) criteria. The diagnostic criteria included infections occurring within 30 days of the procedure, involving at least the skin and subcutaneous tissue, and presenting with one or more of the following: purulent drainage, isolation of an organism from an aseptically obtained culture, or clinical signs such as tenderness, localized swelling, redness, or heat [10].

Data were collected from the paper and electronic (eMedsolution, Zalaegerszeg, Hungary) records of our hospital: medical history, outpatient records, angiographic findings describing the morphology of the affected vessel segment, anesthesia records, detailed surgical records, and results of follow-up examinations. Telephone interviews were conducted with patients and relatives to obtain additional information not available in the hospital records, and to obtain more detailed information about tests and procedures performed in other health care facilities and the current condition of the patients. Our research was conducted under the authorization No. 12/2018 issued by the Semmelweis University Regional and Institutional Committee of Scientific and Research Ethics.

Statistical analyses were performed using Excel (Microsoft Corporation, Redmond, WA, USA), MedCalc^®^ (MedCalc^®^ Statistical Software version 19.6, MedCalc Software Ltd., Ostend, Belgium), and SPSS statistical software v. 24.0 (IBM Corporation, Armonk, NY, USA). Kaplan–Meier survival analysis and the log-rank test were used to determine the time-dependent outcome of overall survival, amputation-free survival, and reintervention-free survival. We used multivariate logistic regression models to provide insight into the relationship between our independent variables (age, smoking, bacteria detected, etc.) and the occurrence of SSI.

## 3. Results

### 3.1. Patient Characteristics

Two hundred thromboendarterectomy procedures with bovine patch angioplasty were performed during the study period; 66% of patients were male, and 34% were female. The median age was 68 (SD ± 9.70) years. The median follow-up was 1010 (SD ± 471.47) days. It was noted that 89% had hypertension, 40% had diabetes, 51% were smokers, 63% had dyslipidemia, and 57% had a cardiovascular event or other cardiovascular disease (e.g., myocardial infarction or ischemic heart disease) before surgery. In addition, 17% had COPD and 22% had a previous vascular surgery in the same region of the body. Clinical presentations were intermittent claudication affecting quality of life (Fontaine IIb, 33%), critical limb ischemia (CLI, 39%) including rest pain (Fontaine III, 12%) and gangrene (Fontaine IV, 27%), acute limb ischemia (18%), and bleeding due to complications following femoral puncture for another intervention (10%). In 46% of cases, FE plus bovine PA was performed without any additional procedures. In 39.5% of cases, FE and PA were combined with transluminal angioplasty. An additional infrainguinal bypass was performed in 5% of cases, and 1% of patients underwent FE with PA combined with infrainguinal bypass and percutaneous transluminal angioplasty (PTA). CFA thromboendarterectomy plus PA was followed by further thrombectomy in 6.5% of the cases studied. Furthermore, 1.5% of the procedures were CERABs (Covered Endovascular Reconstruction of Aortic Bifurcation), where FE and PA were required. Only one patient (0.5%) received a femoro-femoral crossover bypass with FE and PA with further percutaneous transluminal angioplasty. Clinical data of patients are listed in Table 1.

### 3.2. Patency, Reintervention, and Amputation Outcomes

Twenty-five (12.5%) patients had restenosis, and 27 (13.5%) patients had occlusion at the treated site requiring invasive intervention during the follow-up period. Patch rupture was seen in 3% of cases. Wound infection requiring surgery occurred in 32 patients (16%) and typically developed a median of 16 (SD ± 14.48) days after the initial procedure.

**Figure 1:**
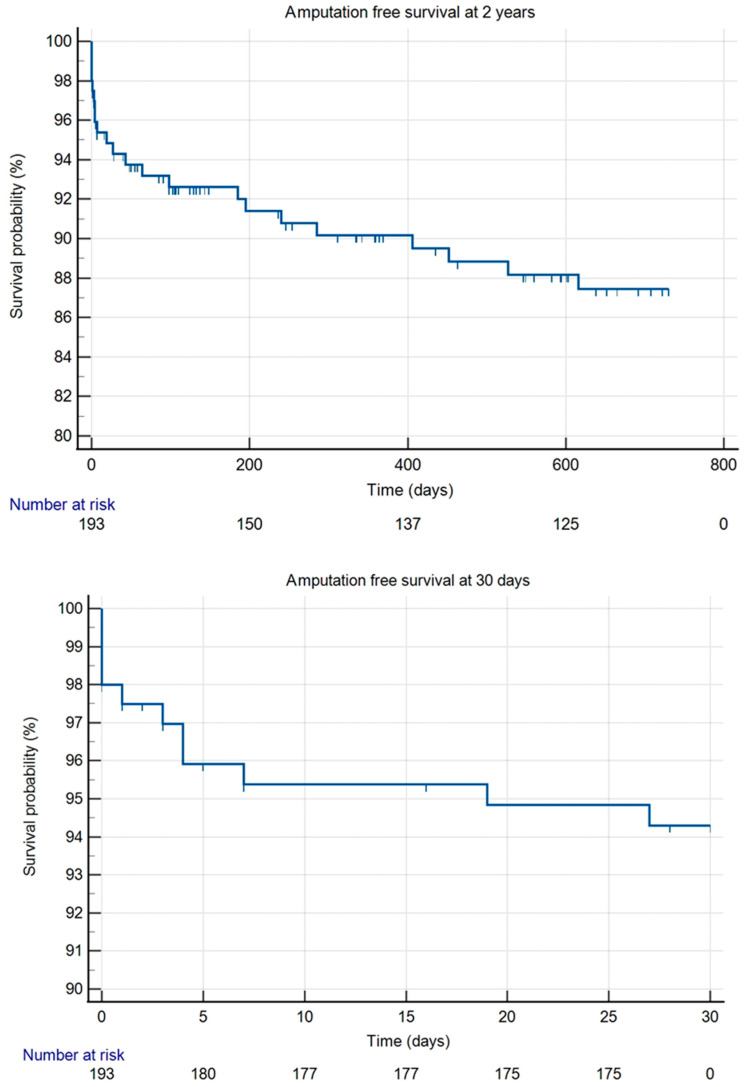
Kaplan–Meier curves for amputation-free survival at 30 days and at 2 years.

**Figure 2:**
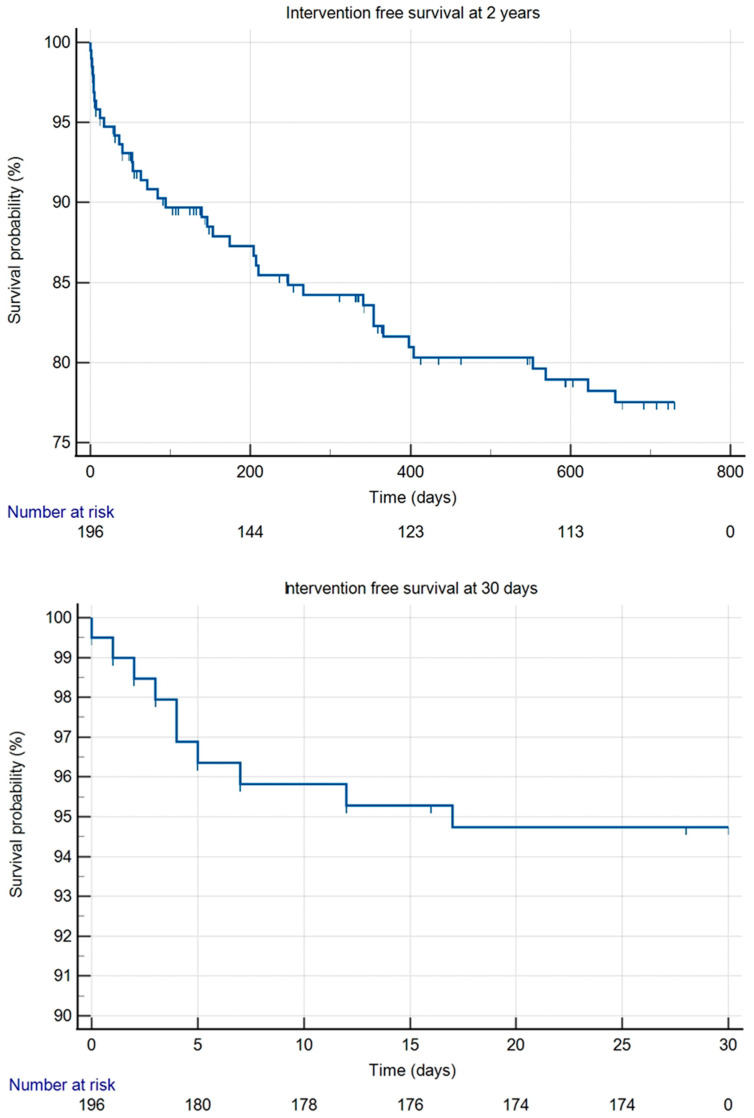
Kaplan–Meier curves for reintervention-free survival at 30 days and at 2 years.

During our follow-up, 88.5% of patients were amputation-free. Seven people had a minor amputation (below the ankle), and 16 people had a major amputation (above the ankle). The amputation-free survival rate was 94.3% at 30 days and 87.4% at 2 years (Figure 1). Reintervention-free survival was 94.7% at 30 days and 77.5% at 2 years (Figure 2).

### 3.3. Survival Outcomes and Causes of Death

**Figure 3:**
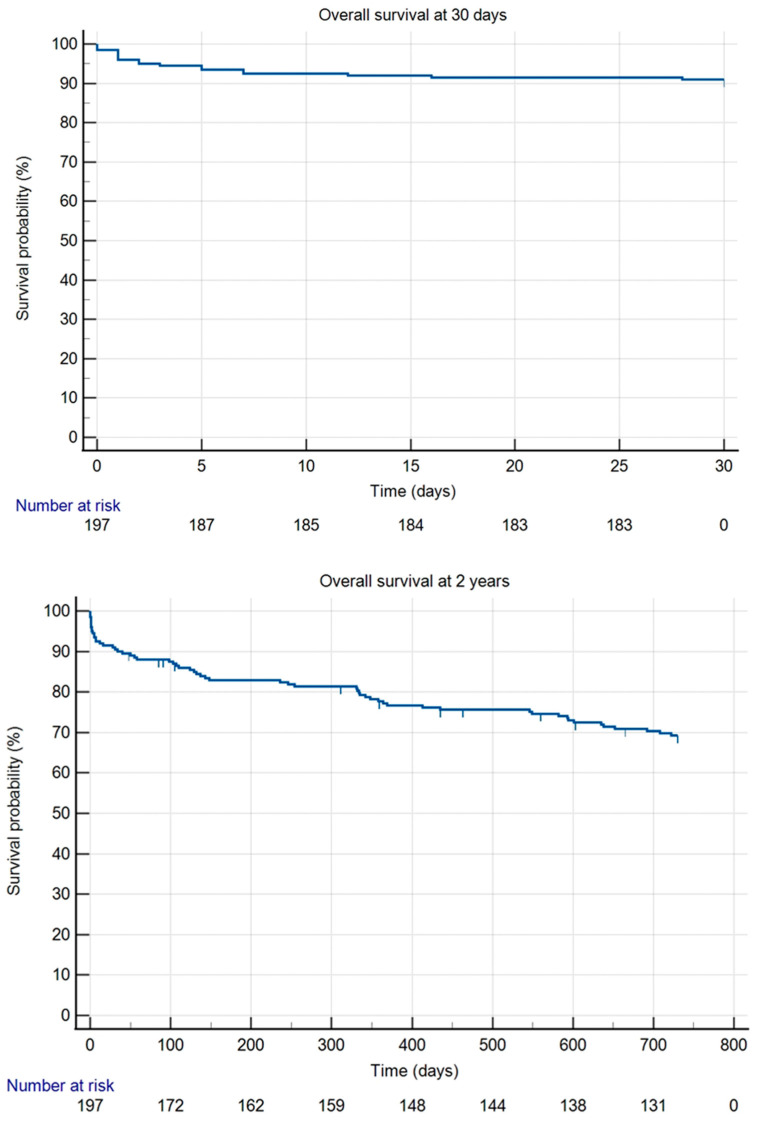
Kaplan–Meier curves for overall survival at 30 days and at 2 years.

Thirty-day survival was 91%, and 2 year survival was 69.3% (Figure 3). The most common cause of death during our follow-up period was heart failure, heart attack, or other cardiovascular causes unrelated to the vascular procedure itself (24.68%). The second most common cause of death was cancer (16.88%). In five cases, the cause of death is unknown (3.9%).

Within the first 30 days, 18 patients died following surgery. Cardiovascular events, including myocardial infarction and heart failure, accounted for 50% of these deaths. Multiorgan failure and sepsis were each responsible for 16.7%, while COVID-19 pneumonia accounted for 11.1%.

During the 2 year follow-up period, 60 patients died. The leading cause of death was cardiovascular events, accounting for 25% of cases. Sepsis was responsible for 20% of deaths, with 11.7% directly related to the vascular reconstruction. Pneumonia accounted for 16.7% of the deaths.

### 3.4. Factors Influencing SSI Development

We obtained bacterial cultures from the surgical site in 67 cases (33.5%) due to the presence of clinical signs of infection, including tenderness, localized swelling, redness, heat, or discharge. In 29 (43.28%) of these patients, no pathogens were found in the sample tested. The most common bacteria found were *Staphylococcus aureus* (14.93%) and *Enterococcus faecalis* (14.93%). Other bacteria detected are shown in Table 2.

Using multivariate logistic regression models, we found that age had no effect on the development of SSI after surgery. While gender did not have a significant effect on developing SSI requiring surgery, we did find that male patients were twice as likely to develop SSI. People with diabetes or a history of cardiovascular disease were more likely to develop SSI, but the differences were not significant. According to our analysis, smoking, hypertension, and previous surgery at the site of the operation did not affect the incidence of SSI.

When analyzing whether the surgical indication influenced the development of SSIs, we found that patients with critical limb ischemia had a 2.04 times higher risk of SSI compared to those with claudication (Fontaine IIb ischemia); however, this association was not statistically significant (*p* = 0.109). Similarly, although not statistically significant (*p* = 0.224), patients with acute limb ischemia appeared to have a 63% lower SSI risk compared to the claudication group. Lastly, patients who underwent FE for bleeding had a 30% lower risk of SSI than those with claudication, but this difference was also not statistically significant (*p* = 0.671).

We examined how specific types of bacteria that colonize the wound affect the development of SSI. We found that the presence of at least one of the following bacteria: methicillin-resistant *Staphylococcus aureus* (MRSA), Escherichia coli, or Pseudomonas aeruginosa was significantly associated with the likelihood of SSI (*p* < 0.001). When at least one of these pathogens was found in the wound, the odds of SSI were 16.1 times higher than when none of these bacteria were present. None of the surgical site infections required removal or replacement of the original bovine pericardium patches; all cases were managed successfully with the patch left in situ. Key metrics are listed in Table 3 and Table 4.

## 4. Discussion

### 4.1. Bovine Pericardium Patch: Advantages and Current Evidence

The bovine patch is a preferred material alongside venous patches and has been used for many years due to its specific favorable properties, including resistance to infection, minimal bleeding from suture holes, and lower thrombogenic potential compared to synthetic patch materials [5]. BPP also offers a durable alternative to the saphenous vein for arterial reconstruction after the removal of an infected arterial graft [11]. The follow-up reports of BPP usage in infection-free common femoral arteries are scarce [12,13].

BPP has a low rate of patch rupture, although other studies using venous patches have reported rupture rates similar to our findings with bovine patches [12,14].

One of the critical issues with FE with BPP is the high rate of postoperative complications. In the CAULIFLOWER study, postoperative complications were more common with FE than with endovascular approaches. However, it is important to note that in this study they used venous or synthetic patch materials such as polytetrafluoroethylene and Dacron. According to the CAULIFLOWER data, FE had significantly higher rates of primary patency and freedom from reintervention compared to endovascular therapies [15]. A meta-analysis by Boufi et al. also reported that the long-term primary patency rate was much higher after open surgery [16].

### 4.2. Patency, Reintervention, and Amputation-Free Survival

We evaluated the reintervention-free survival rates after bovine patch angioplasty at our center. In this study, we evaluated the reintervention-free survival rates specifically at the surgical site following bovine patch angioplasty. Although our results show relatively good primary patency rates, they are at the lower end of those reported in other centers (80–98%) [12,13,17,18]. We also reported lower limb salvage rates at 2 years [13,19]. Significant aspects are stages of atherosclerosis and the demographic and clinical profile of our patient population. Patients treated at our center were more likely to have comorbidities such as diabetes and cardiovascular disease. These conditions are well-established risk factors for precipitating the major life-threatening events in atherosclerosis, including limb ischemia. Diabetes also plays a major factor in delayed wound healing and restenosis following vascular interventions [20,21,22]. In addition, a higher proportion of patients in our patient population were smokers compared to those reported in other studies. Smoking is one of the most detrimental factors contributing to vascular dysfunction, as it promotes vasoconstriction and exacerbates vascular oxidative stress, leading to endothelial damage [23]. According to a retrospective cohort study, smoking was also associated with a significantly increased risk of postoperative wound disruption and SSI [24]. These persistent harmful effects may explain the differences in patency outcomes observed in our study.

### 4.3. Surgical Site Infections (SSI): Incidence and Risk Factors

Surgical site infections have always been reported in other studies in the range of 14–17%, which is similar to our findings [25,26,27]. The high prevalence of cardiovascular disease and diabetes in patients with CLI in our cohort suggests that CLI is a marker of systemic vascular disease. This is consistent with the finding that CLI is associated with an increased risk of SSI, as these comorbidities impair wound healing and the immune response. In their cohort study, Langenberg et al. also reported a trend towards the influence of diabetes mellitus on the development of SSI, but similar to our findings, this was not statistically significant. They also found that patients who developed an SSI had a significantly longer hospital stay, averaging 20 days [28]. A systematic review and meta-analysis also identified CLI as a risk factor for increased development of SSI after lower limb revascularization surgery [29]. According to Dosluoglu et al., the duration of surgery does not seem to play an important role in the development of SSI, as they reported lower SSI rates after hybrid surgery than after open endarterectomy [30]. However, another study suggests that longer procedure times are associated with an increased rate of SSI [21].

### 4.4. Role of Bacterial Colonization and SSI Prevention Strategies

Our result highlights the importance of monitoring and managing infections caused by MRSA, *E. coli* and *P. aeruginosa* bacteria in surgical wounds. While a variety of pathogens can contribute to SSIs, including fungi and bacteria, the literature suggests that *P. aeruginosa*, *S. epidermidis,* and *S. aureus* strains are most commonly associated with SSIs [21,22,31,32,33]. The bacterial typology of vascular SSI has changed over time and is reflected in an increased incidence of antibiotic-resistant bacteria, particularly members of the Staphylococcus family, in particular, methicillin-resistant *Staphylococcus aureus* (MRSA) [31]. Proactive measures (e.g., preoperative antibiotic prophylaxis, enhanced sterilization protocols, or targeted infection control strategies) may help reduce the risk of SSI associated with these pathogens [34,35,36]. Although routine use of vancomycin prophylaxis is generally not recommended, vancomycin may be used for prophylaxis in hospitals with high MRSA prevalence or in patients at high-risk of infection (e.g., geriatric, oncological, and dialysis patients) [37]. Administration of local vancomycin to inguinal wounds did not decrease the rate of deep SSIs, but it had a positive effect on superficial wound infections [38]. Decolonization of *S. aureus* with nasal mupirocin combined with chlorhexidine body wash prior to vascular surgery reduces the incidence of surgical site infection caused by *S. aureus* in patients who are *S. aureus* carriers [39]. Costa Almeida et al. used Gentamicin Collagen Implants (GCCI) in the inguinal incision adjacent to the prosthesis and found that the use of GCCI reduced the SSI rate and the number of days spent in the hospital. However, the limitation of this study is that they only included non-diabetic and non-obese patients [40]. It is important to note that local antibiotics are not routinely used today because of the lack of evidence.

### 4.5. Study Limitations

While this study provides important real-world insights, several limitations should be considered when interpreting the findings. First, its retrospective, single-center design may limit the broader applicability of the results. Second, due to the real-world nature of the cohort, a substantial number of patients underwent concomitant procedures—such as percutaneous transluminal angioplasty, infrainguinal bypass, or thrombectomy—which may have influenced key outcomes such as patency and reintervention rates. We did not perform subgroup analyses to isolate the effects of BPP use in standalone femoral endarterectomy, which may introduce confounding. Third, we did not collect detailed data on the patency of runoff vessels distal to the common femoral artery, such as the deep and superficial femoral artery or popliteal-crural vessels, which may significantly impact limb salvage and graft durability. Additionally, the bacteriological data may be incomplete, as cultures were only obtained in suspected infections, and follow-up imaging was not standardized across all patients. Finally, although the length of median follow-up was substantial, longer-term outcomes beyond 2 years would provide more robust insights into durability and late complications. Future prospective studies with standardized follow-up protocols and stratification of adjunctive procedures are warranted to validate these findings.

## 5. Conclusions

Our findings suggest that bovine pericardium patch is safe and effective for common femoral artery endarterectomy. Differences in patient characteristics, such as a higher prevalence of comorbidities and smoking, are likely to contribute to the variability in outcomes between centers. Our high patency and low reinfection rates highlight the effectiveness of bovine patch angioplasty even in a high-risk patient population. Management of patient comorbidities and optimization of infection prevention strategies are essential to improve long-term outcomes.

## Figures and Tables

**Table 1:** Clinical data of patients. ^1^ CVD: cardiovascular disease. ^2^ COPD: chronic obstructive pulmonary disease. ^3^ ALI: acute limb ischemia. ^4^ FE: femoral endarterectomy. ^5^ PA: patch angioplasty. ^6^ PTA: percutaneous transluminal angioplasty. ^7^ CERAB: covered endovascular reconstruction of aortic bifurcation. ^8^ SSI: surgical site infections. ^9^ MOF: multiorgan failure.

Clinical Characteristics	Number of Patients (%)
Hypertension	178 (89)
Diabetes mellitus	79 (40)
Smoking	102 (51)
Dyslipidemia	125 (63)
CVD ^1^	114 (57)
COPD ^2^	33 (17)
Previous surgery	44 (22)
Indication for revascularization	
Intermittent claudication (Fontaine IIb)	66 (33)
Rest pain (Fontaine III)	24 (12)
Gangrene (Fontaine IV)	54 (27)
ALI ^3^	36 (18)
Bleeding	20 (10)
Type of revascularization	
FE ^4^ + PA	92 (46)
FE + PA ^5^ + PTA ^6^	79 (39.5)
FE + PA+ infrainguinal bypass	10 (5)
FE + PA infrainguinal bypass + PTA	2 (1)
FE + PA + thrombectomy	13 (6.5)
FE + PA + CERAB ^7^	3 (1.5)
FE + PA + femoro-femoral crossover bypass + PTA	1 (0.5)
Postoperative complications	
Restenosis requiring surgery	25 (12.5)
Occlusion requiring surgery	27 (13.5)
Patch rupture	6 (3)
SSI ^8^ requiring surgery	32 (16)
Major amputation	16 (8)
Minor amputation	7 (3.5)
Cause of death	
Tumor	13 (16.88)
Pneumonia, respiratory failure	5 (6.49)
MOF ^9^	5 (6.49)
Sepsis	6 (7.79)
Direct cause of the vascular surgery	9 (11.69)
Cardiovascular cause unrelated to the surgery	19 (24.68)
Gastrointestinal	6 (7.79)
COVID-19 infection	5 (6.49)
Stroke	3 (3.9)
Other (known cause but can not be classified)	1 (1.3)
Unknown	5 (6.49)

**Table 2:** Detected bacteria from cultures. ^1^ MRSA: methicillin-resistant *Staphylococcus aureus*.

Bacteria Detected from Cultures	Number of Patients (%)
No bacteria	29 (43.28)
*S. aureus*	10 (14.93)
MRSA ^1^	4 (5.97)
*E. coli*	5 (7.46)
*S. agalactiae*	3 (4.48)
Proteus species	5 (7.46)
*E. cloacae*	5 (7.46)
*C. perfringens*	1 (1.49)
*E. faecalis*	10 (14.39)
*P. aeruginosa*	5 (7.46)
*S. epidermidis*	4 (5.97)
Klebsiella species	2 (2.99)
*Campylobacter ureolyticus*	1 (1.49)
*S. haemolyticus*	1 (1.49)
Salmonella species	1 (1.49)

**Table 3:** Influence of demographic factors, comorbidities, and bacterial colonization on surgical site infection (SSI): logistic regression analysis. ^1^ CVD: cardiovascular disease; ^2^ MRSA: methicillin-resistant *Staphylococcus aureus*.

Variables	S.E.	Sig. (*p*-Value)	Exp(B) (Odds Ratio)	95% CI for Exp (B)
Age	0.020	0.653	0.991	0.95–1.03
Gender	0.428	0.053	2.540	0.99–6.50
Diabetes	0.388	0.353	1.434	0.67–3.07
Smoking	0.386	0.611	0.822	0.39–1.75
Hypertension	0.590	0.768	0.840	0.26–2.67
CVD ^1^	0.397	0.494	1.312	0.60–2.90
Previous surgery	0.564	0.165	0.457	0.15–1.38
MRSA ^2^, *E. coli*, *P. aeruginosa*	0.620	<0.001	16.1	4.775–54.23

**Table 4:** Association between surgical indication and risk of surgical site infection (SSI): logistic regression analysis. ^1^ CLI: critical limb ischemia; ^2^ ALI: acute limb ischemia.

Indication for surgery	S.E.	Sig. (*p*-Value)	Exp (B) (Odds Ratio)
Claudication		0.071	
CLI ^1^	0.445	0.109	2.040
ALI ^2^	0.811	0.224	0.373
Bleeding	0.827	0.671	0.704
Constant	0.359	0.000	0.158

## Data Availability

The data presented in this study are available on request from the corresponding author due to privacy and ethical reasons.

## Abbreviations

The following abbreviations are used in this manuscript:

ALI	Acute limb ischemia
AVPs	Autologous vein patches
BPP	Bovine pericardial patch
CDC	Centers for Disease Control and Prevention
CERAB	Covered Endovascular Reconstruction of Aortic Bifurcation
CFA	Common femoral artery
CFE	Common femoral endarterectomy
CLI	Chronic limb ischemia
COPD	Chronic obstructive pulmonary disease
CTA	Computed tomography angiography
CVD	Cardiovascular disease
DSA	Digital subtraction angiography
FE	Femoral endarterectomy
MOF	Multiorgan failure
MRSA	Methicillin-resistant *Staphylococcus aureus*
PA	Patch angioplasty
PTA	Percutaneous transluminal angioplasty
